# The influence of riverine barriers, climate, and topography on the biogeographic regionalization of Amazonian anurans

**DOI:** 10.1038/s41598-018-21879-9

**Published:** 2018-02-21

**Authors:** Marcela Brasil de Castro Godinho, Fernando Rodrigues da Silva

**Affiliations:** 1 Programa de Pós-Graduação em Biologia Animal, Universidade Estadual Paulista Júlio de Mesquita Filho – UNESP, campus de São José do Rio Preto, São Paulo, 15054-000 Brazil; 2 Laboratório de Ecologia Teórica: Integrando Tempo, Biologia e Espaço (LET.IT.BE), Departamento de Ciências Ambientais, Universidade Federal de São Carlos, campus Sorocaba, São Paulo, 18052-780 Brazil

## Abstract

We evaluated five non-mutually exclusive hypotheses driving the biogeographic regions of anuran species in the Amazonia. We overlaid extent-of-occurrence maps for anurans 50 × 50 km cells to generate a presence–absence matrix. This matrix was subjected to a cluster analysis to identify the pattern and number of biogeographic regions for the dataset. Then, we used multinomial logistic regression models and deviance partitioning to explore the relative importance of contemporary and historical climate variables, topographic complexity, riverine barriers and vegetation structure in explaining the biogeographic regions identified. We found seven biogeographic regions for anurans in the Amazonia. The major rivers in the Amazonia made the largest contribution to explaining the variability in anuran biogeographic regions, followed by climate variables and topography. The barrier effect seems to be strong for some rivers, such as the Amazon and Madeira, but other Amazonia rivers appear to not be effective barriers. Furthermore, climate and topographical variables provide an environmental gradient driving the species richness and anuran range-size distributions. Therefore, our results provide a spatially explicit framework that could be used to address conservation and management issues of anuran diversity for the largest tropical forests in the world.

## Introduction

The spatial patterns of species distributions express many ecological and evolutionary processes and are linked to a complex and historically contingent setting. Since the 19th century, studies have divided large geographic extents into regions of similar faunistic or floristic composition^1–4^. This approach, called biogeographical regionalization, has helped us understanding whether the processes influencing species distributions are determined by shared evolutionary histories (i.e. speciation, extinction and distribution), past or current climatic oscillations (i.e. precipitation and temperature gradients) and/or physical barriers (i.e. mountains and oceans) that limit species dispersal between areas^2,4–8^. For example, Holt *et al*.^2^ identified 20 distinct zoogeographic regions by combining data on the distributions and phylogenetic relationships of vertebrate species and found that spatial turnover in phylogenetic composition is higher in the Southern than in the Northern Hemisphere. Furthermore, global biogeographical regionalization has been used to evaluate international conservation priorities based on the degradation of natural habitats and ecosystems as a result of human activities^3,9^. Although large-scale global patterns are relatively well established^2,4,9^, intracontinental regionalization patterns are still scarce for some Neotropical areas, representing an opportunity for new insights about the processes influencing species distributions^8,10^.

The Amazonia encompasses more than 6 million km^2^ across eight countries in South America and is one of the most critical natural environments both in regulation climate and sustaining biodiversity at global scale^11,12^. Currently, Amazonia is threatened by several anthropic pressures, such as dam constructions, deforestation, and fire that will cascade onto the patterns of species distribution of the largest and most species-rich tropical forest in the world. Although, previous studies have delimited biogeographical regions for mammals and birds in the Amazonia, their results are not consensual. For example, Wallace^1^, considering primate ranges, identified four regions in the Amazonia. Haffer^13^, Cracraft^14^ and Silva *et al*.^15^, considering bird ranges, identified six, seven and eight regions in the Amazonia respectively. Thus, uncertainty about biogeographical regionalization in the Amazonia remains open to debate and different hypotheses have been proposed to explain the pattern of species distributions in the Amazonia^1,16–19^. Among competing hypotheses, the riverine barrier hypothesis states that the major rivers of Amazonia act as geographic barriers to gene flow, promoting the genetic divergence of populations and, therefore, speciation^1^. The Pleistocene refuge hypothesis states that during the Pleistocene, decreases in temperature and humidity in the Amazonia Basin left relatively small ‘islands’ of tropical rainforests surrounded by xeric habitats, isolating populations and changing distribution patterns^4,16^. The orogenic hypothesis states that the uplift of the Andes in Neogene and its effect on regional climate has had a substantial impact on the landscape evolution in the Amazonia^18^. Therefore, the pattern of species distribution in Amazonia will not be explained entirely by any single simple model, but it depends on the combination of more realistic, complex scenarios^19–21^.

Biogeographical units are hierarchically arranged, and no single biogeographic framework is optimal for all taxa^2,3,6,9^. To date, no study has evaluated the importance of multiple scenarios shaping present-day patterns of amphibian species composition along the Amazonia. Amphibians are the most threatened vertebrate group^22^, with Amazonia harboring the highest species richness in the world^23^. Moreover, patterns of amphibian species richness distribution are not randomly distributed throughout the Amazonia^23,24^. Because amphibian species are normally separated into more regions than other vertebrate groups due to their small-ranges^25^ and physiological constraints^6,26^, we believe that the Amazonia will present more than the eight regions previously proposed for birds^14,15^. Here, we performed a regionalization scheme for the current original extent of the Amazonia in order to explore how anurans are distributed throughout this complex and biodiverse biome. Our goal is to determine the biogeographical regions for anuran species in the Amazonia evaluating five non-mutually exclusive hypotheses:

i) *Contemporary Climate hypothesis* – present-day climate variables are key environmental determinants of anuran composition because they act as environmental filters influencing which species can inhabit specific areas^26,27^. Under this hypothesis is expected that areas with different climate gradients would harbor distinct species compositions due to specific physiological requirements or life history traits;
ii) *Pleistocene Climate Variation hypothesis* – while current patterns of amphibian distributions in Europe^28^ and Brazilian Atlantic Forest^29^ were shaped by climate changes in the past, there is still no evidence that amphibian distributions in Amazonia has been influenced by Pleistocene climate variation^30^. However, Amazonia presents a large spatial extent, and stable climatically areas since the Pleistocene were not randomly distributed in the space^15^. Under this hypothesis is expected that areas that maintained similar climatic conditions, but are far apart from each other, would harbor distinct species compositions due to dissimilar rates of speciation, extinction and colonization that delimited different regional species pools^31,32^ along the Amazonia;
iii) *Topography hypothesis* – areas with larger ranges in elevation increase the speciation rate and endemism^18,33^. Under this hypothesis is expected that these areas would harbor small-ranged species with historically limited dispersal capabilities due to physical barriers and/or physiological constraints;
iv) *Vegetation Structure hypothesis* – the concept of habitat templets argues that habitat provides the templet on which evolution forges animal life-history strategies. Based on this idea, previous studies have found that floristic structure has a strong correlation with the biogeographic regions of amphibians identified in Europe^6^ and the Brazilian Atlantic Forest^8,10^. Under this hypothesis is expected that the biographical regions of anurans would be recognized because of the vegetation distribution within the Amazonia;
v) *Riverine Barrier hypothesis* – the major rivers of Amazonia act as geographic barriers to the dispersal of organisms and hamper gene flow between populations, increasing speciation rates^1,12,34^. Under this hypothesis is expected that some anuran species could not traverse the major rivers in the Amazonia, thus creating different species compositions between opposite banks of the major rivers of Amazonia.

## Results

**Figure 1:**
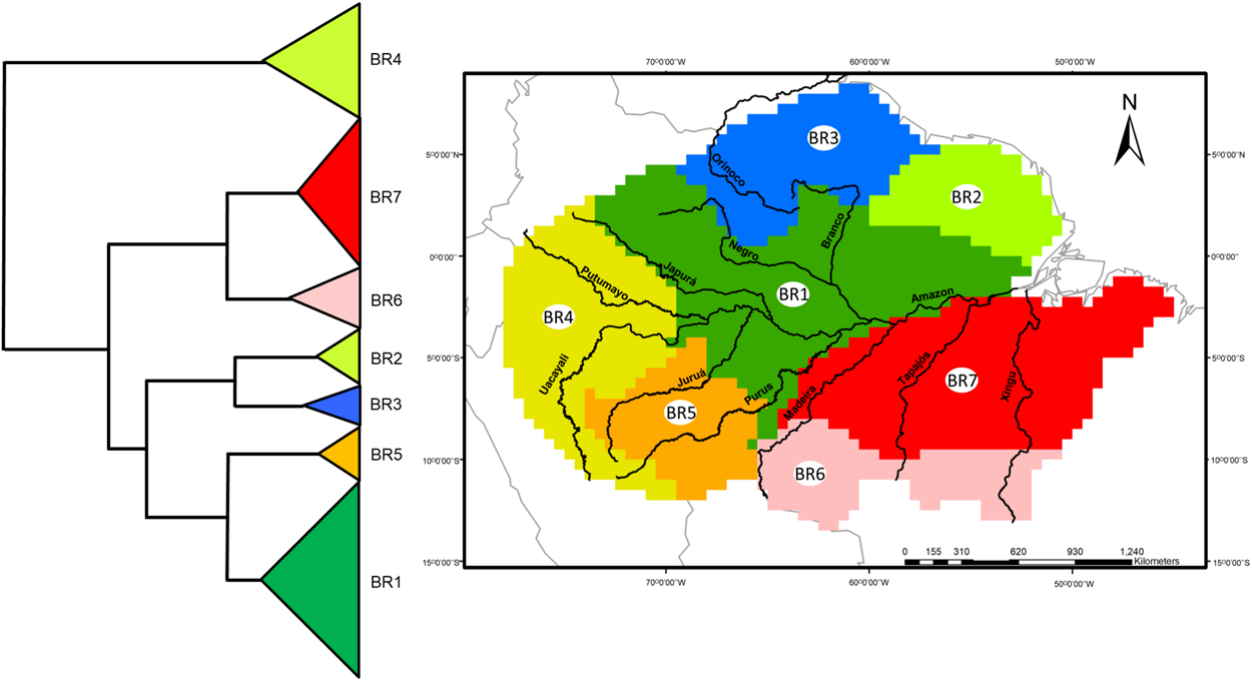
Dendrogram and Amazonia map depicting the regionalization of anuran dissimilarity into seven biogeographical regions (BR) based on *recluster*.*region* algorithm^68^. Colors used in dendrogram and map are identical. Black lines in the map represent the ten major rivers in Amazonia. Map generated using ESRI ArcMap 9.2. https://www.esri.com.

**Figure 2:**
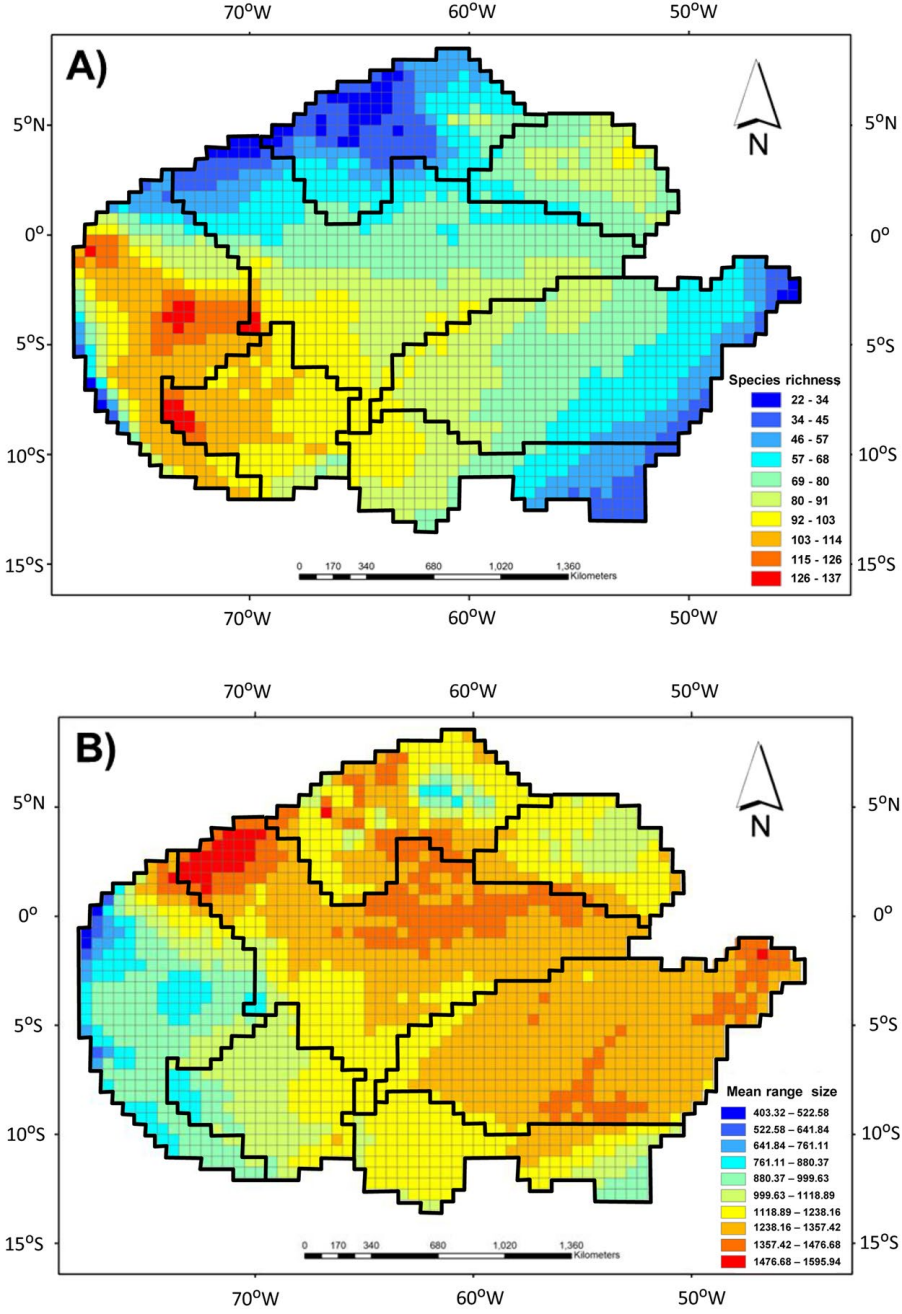
Gradients of (**A**) anuran species richness, and (**B**) mean range-size of anuran species occurring in each grid of the Amazonia. Black lines are delimiting the seven biogeographical regions of anuran identified in Fig. 1. Maps generated using ESRI ArcMap 9.2. https://www.esri.com/.

We identified seven biogeographic regions in the Amazonia based on anuran species composition with explained dissimilarity values of 92% and a mean silhouette width of 0.33 (Fig. 1, Table 1). From the seven biogeographic regions observed, three biogeographical regions (BR1, BR2, and BR3) are delimited to the north of the Amazon River, three (BR5, BR6, and BR7) are delimited to the south of the Amazon River and one (BR4) is delimited in the western portion of Amazonia (Fig. 1). The grids in BR4 contain the highest values of species richness, while the grids in BR3, BR6, and BR7 contain the lowest values (Fig. 2A). Based on the range size of species distributions, we observed that BR4 contains anuran species with restricted range sizes while the grids in the BR1 and BR7 contain anuran species with wide range sizes (Fig. 2B).

**Table 1:** Values of the mean silhouette width (Silh) and the explained dissimilarity (ex.diss) for all the clustering solutions.

Number of cluster	Silh	ex.diss	Number of cluster	Silh	ex.diss
2	0.410	0.384	27	0.351	0.990
3	0.290	0.707	28	0.355	0.991
4	0.314	0.840	29	0.359	0.991
5	0.298	0.876	30	0.363	0.992
6	0.313	0.909	31	0.369	0.992
**7**	**0.336**	**0.925**	32	0.370	0.993
8	0.334	0.937	33	0.378	0.993
9	0.346	0.942	34	0.371	0.993
10	0.320	0.953	35	0.374	0.994
11	0.319	0.960	36	0.376	0.994
12	0.321	0.966	37	0.363	0.994
13	0.335	0.968	38	0.357	0.994
14	0.355	0.971	39	0.352	0.994
15	0.340	0.975	40	0.346	0.994
16	0.340	0.979	41	0.352	0.995
17	0.344	0.980	42	0.356	0.995
18	0.348	0.981	43	0.361	0.995
19	0.337	0.983	44	0.362	0.995
20	0.354	0.984	45	0.364	0.996
21	0.342	0.985	46	0.360	0.996
22	0.350	0.986	47	0.363	0.996
23	0.326	0.987	48	0.365	0.996
24	0.346	0.988	49	0.364	0.996
25	0.333	0.989	50	0.366	0.996
26	0.348	0.990			

The mean silhouette width measures the strength of any of the partitions of objects from a dissimilarity matrix^70^.The explained dissimilarity maximizes between-cluster variation relative to within-cluster variation^2^. In bold the number of cluster selected.

**Figure 3:**
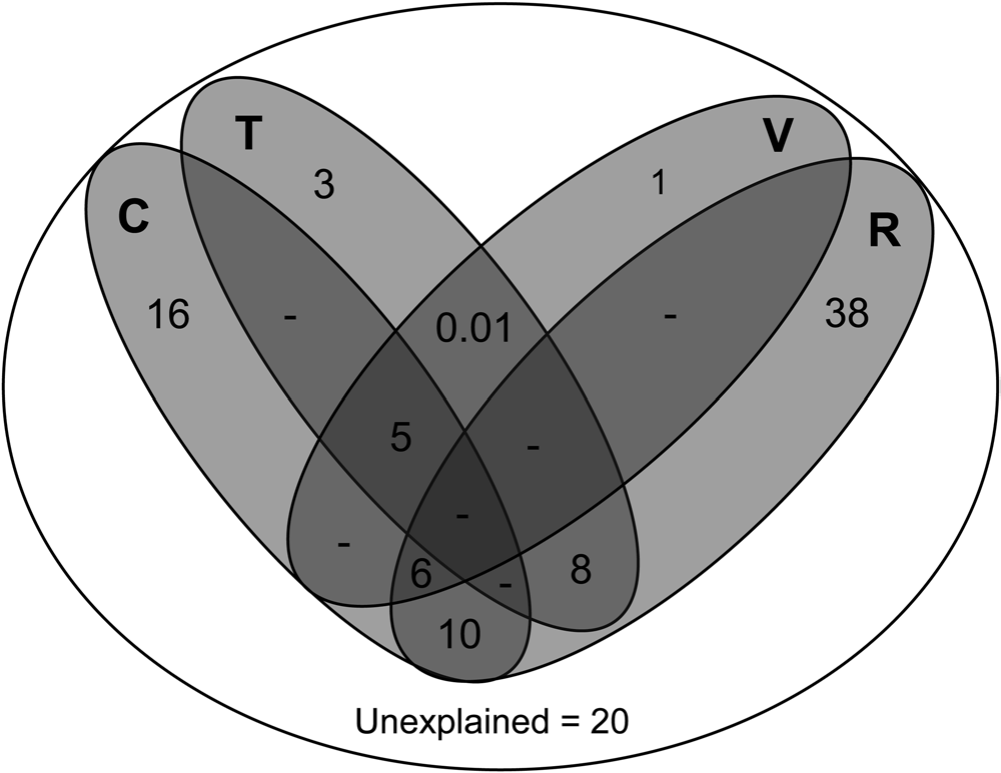
Partitioning analysis representing the deviance in the biogeographic regions configurations explained by climate (current + historical difference), topography, riverine barriers and vegetation structure of the Amazonia. C = climate, T = topography, V = vegetation structure, R = riverine barriers.

Among all models of predictor variables, the model without VEGE.PC1 and TOPO.PC2 was the best one for explaining the cluster patterns (∆ AICc > 6.4; Table 2). This model explains 80% of the cluster patterns (Table 2). The partitions of deviance indicated that the independent effect of riverine barriers accounted for 38% of the variability in the anuran biogeographic regions, followed by climate variables with 16% and topography with 3% (Fig. 3). Vegetation structure has a weak association with anuran biogeographic regions in the Amazonia (Fig. 3).

**Table 2:** The six most parsimonious multinomial logistic regression models used to investigate the influence of current (CURE.PC1 and CURE.PC2) and Pleistocene (HDT and HDP) climate conditions, topography (TOPO.PC1 and TOPO.PC2), riverine barriers (RIVERS) and vegetation structure (VEGE.PC1 and VEGE.PC2) in explaining the biogeographical regions for anurans in the Amazonia.

Models	ΔAICc	df	wAICc	%DE
CURE.PC1 + CURE.PC2 + HDT + HDP + TOPO.PC1 + RIVERS + VEGE.PC2	0	96	0.95	80.3
CURE.PC1 + CURE.PC2 + HDT + HDP + TOPO.PC1 + RIVERS + VEGE.PC1 + VEGE.PC2	6.49	102	0.03	80.2
CURE.PC1 + CURE.PC2 + HDT + HDP + TOPO.PC1 + TOPO.PC2 + RIVERS + VEGE.PC2	9.41	102	0.009	79.9
FULL MODEL	14.06	108	0.001	81
CURE.PC1 + CURE.PC2 + HDT + HDP + TOPO.PC1 + RIVERS + VEGE.PC1 + VEGE + PC2	25.72	96	<0.001	79.8
CURE.PC1 + CURE.PC2 + HDT + TOPO.PC1 + RIVERS + VEGE.PC2	32.36	90	<0.001	77.7

ΔAICc = difference between the interest model and the model with the lowest value of Akaike information criterion correct for small samples; wAICc = AICc weight model that expresses the weight of evidence favoring the model as the best among all the models compared; %DE = percent deviance explained in the response variable by the model under consideration.

## Discussion

This is the first study showing that multiple factors shape anuran biogeographical regions in Amazonia. We found that the major rivers in Amazonia strongly contributed to explaining the variability in anuran biogeographic regions, followed by climate and topography variables. We identified seven biogeographic regions that partially overlap with the eight areas of endemism previously proposed for terrestrial vertebrates in Amazonia^14,15^. To the north of the Amazon River, we found that part of BR1, BR2, and BR3 are nested in the area corresponding to Guiana, a biogeographic unit identified by Cracraft^14^. BR4 and BR5 are partially congruent with the areas of Napo and Inambari, identified by Cracraft^14^, respectively. However, BR6 and BR7 in the southern Amazon River differ from spatial arrangements of the Rondonia, Tapajós, Xingu, and Belém areas of Cracraft^14^ and Silva *et al*.^15^. There is no single biogeographic solution that is optimal for all taxa^1,2,4,7,14,15^. For example, Rueda *et al*.^6^ found substantial variation in the number of regions considering different taxonomic groups in Europe. Naka^20^, redefined the boundaries of the Guiana region^14^ for Amazonia birds using different quantitative methods. Therefore, regionalization patterns depend on the taxonomic group of interest or the clustering methods used to delineate biogeographic units^7,35^. Because previous studies in Amazonia were performed with the spatial distributions of primate^1^ and bird^14,15,20^ species, our results provide new information about the factors associated with the spatial patterns of anuran species distribution in Amazonia.

A cluster analysis based on amphibian distribution recognized the boundaries of Amazonia as one of the four biogeographic regions in South America^27^. We scaled down the analysis and described the effects of riverine barriers, climatic and topographic variables acting inside Amazonia. Our results showed that the Amazon River separates the biogeographical regions in the north (BR1, BR2, and BR3) from those in the south (BR5, BR6 and BR7), while the Madeira river separates the southeastern biogeographical regions (BR6 and BR7) from that in the southwest (BR5). A longstanding debate exists as to whether the riverine barrier hypothesis has played an important role in shaping the present-day species distribution patterns in Amazonia^1,14,19,21,33,34,36^. Wallace^1^ defined distinct areas based on primate species composition that were separated by the Amazon, Solimões, Negro, and Madeira rivers. Recently, Dias-Terceiro *et al*.^37^ and Moraes *et al*.^34^ showed that the Madeira and Tapajós River respectively are barriers to some amphibian lineages in western and eastern Amazonia. In contrast, Gascon *et al*.^33^ did not find a relationship between amphibian species composition and the banks of the Juruá River. Taken together, these results indicate that rivers contribute unequally to the observed patterns of amphibian distribution in the Amazonia. Oliveira *et al*.^21^ found similar results to bird distributions and showed that some bird species with low dispersal ability were limited by all major Amazonia rivers, while many other species can apparently cross some rivers. Thus, the barrier effect might be strong for some rivers, such as the Amazon and Madeira, but others rivers might not be an effective barrier. We still lack a consensus on why different rivers are barriers to some species of mammals, birds, and amphibians but not others. To improve our understanding, we must consider life-history traits, dispersal ability, and phylogenetic relationships that are undoubtedly important factors related to the patterns of species distributions^21,34^. However, considering that over 2,000 new species of plants and vertebrates having been described since 1999^12^, several of these information are currently lacking for most of species in the Amazonia.

Climate and topographic variables explained the second and third highest percentages of variance in the distribution of biogeographic regions, respectively. This result agrees with previous studies that defined biogeographic regions for amphibians in South America^27^, Europe^6^, the Atlantic Forest^8^ and at a global scale^4^. Amazonia has a well-defined climate gradient, with southeastern areas presenting warmer and more seasonal climate than northwestern areas^11,18^. This pattern is associated with orography of the northwestern areas, which contain the highest elevations in the Amazonia. We found that most small-ranged anuran species inhabit biogeographic regions with high elevations and humidity. Mountains affect species richness by fostering the diversification of unique lineages and as natural barriers to species with limited dispersal ability^38,39^. The distribution of amphibian species richness is usually associated with physiological constraints that reflects differences in tolerance to precipitation and temperature^38,40–42^. For example, Da Silva *et al*.^26^ found that humidity-related variables are key environmental factors related to both the richness of reproductive modes and anuran phylogenetic diversity in the Brazilian Atlantic Forest. Variation in the climatic and orographic variables seem to influence speciation, extinction, and dispersal rates of anuran species throughout Amazonia^43,44^. Therefore, different from Ficetola *et al*.^4^ who found that continental drift, climate differences, and mountain chains interact to determine the boundaries of biogeographic regions at global scale, we highlight an important role for climatic and orographic variables shaping anuran distributions at intermediate scale.

Previous studies have found that vegetation structure is an important factor related to biogeographical regions for amphibians^6,8^. In contrast, we found that vegetation types have a weak association with biogeographical regions. According to Charity *et al*.^12^, moist forest is the dominant vegetation type in the Amazonia, covering nearly 80 percent of the biome; other forest types include flooded and swamp forests (3.9 per cent), deciduous forest (1.4 per cent), savannah (6.8 per cent) and others (1.1. per cent). At broad scales, this homogenization of vegetation decreases the importance of vegetation structure in explaining the distribution of biogeographical regions. However, this is not the case when considering finer scales. For example, Gascon *et al*.^33^ found that flooded versus upland forest is an important predictor of community similarity in species composition of amphibians at the Juruá River. Islands of savannah of varying size occurring within the Amazonia biome are home to unique flora and fauna, including numerous endemics. Nonetheless, Amazonia savannahs are little known, highly threatened, and under-protected^45^. Thus, vegetation structure might be important for the distribution of biodiversity and conservation purposes when evaluating the biogegraphical units at finer resolutions.

For the first time, BR1 appears as a biogeographic region in the central part of Amazonia. One possible explanation for the identification of BR1 is that it is a biogeographical transition zone, representing geographical areas of species overlap, with a gradient of replacement and partial segregation between anuran species from neighboring biogeographic regions creating a distinct species composition^46,47^. Biogeographical transition zone is an area where historical and ecological changes allow both the mixture and the co-occurrence of species from two or more biogegraphical regions^46^. For example, the boundaries of BR1 are in contact with those of six biogeographic regions. If BR1 shares some anuran species with each of the six neighboring biogeographic regions, its identification as a biogeographic transition zone is valid. However, our knowledge of biodiveristy distribution is far from complete, and the geographical distribution of species already described is also fragmentary (i.e. Wallacean shortfall^48^). We are aware that the accuracy of amphibian range maps is not without criticism, mainly in megadiverse tropical regions, such as Amazonia^49^. Thus, the identification of BR1 could also be an artefact of the limitation in the knowledge about anuran distribution^49^. For example, Naka^20^ found a single area of endemism for 85 avian species in the Guiana shield that coincides with part of our BR1 and BR2 boundaries. This area of endemism is congruent with the Amazon River to the south, the lower Negro river to the south-west, and the Branco river to the west^20^. The remaining part of BR1 are congruent with the area of Imeri identified by Cracraft^14^. This area of endemism is congruent with the Negro River to the north-east and the Japurá river to the south-west^14^. Therefore, future studies with more accurate information on anuran distribution in Amazonia will be able to answer whether BR1 is a valid biogeographic region or an artifact of limited current datasets.

Biogeographical regionalization provides a framework for addressing evolutionary and ecological processes that underlie present-day distributions and several studies have used them as templates to test areas of endemism, historical relationship among areas, delimit regional species pools, and investigate macroecological patterns^5,7,9,31,47^. Understanding the occurrence of different species in particular geographical areas permit the identification of patterns that can be the starting point in conservation biogeography^50,51^. For example, the frog-killing fungus *Batrachochytrium dendrobatidis*, has been linked to extirpations and extinctions of amphibian species in several continents^52^ and one of the main hypotheses explaining this decline is the side effects of climate change^53,54^. Becker *et al*.^55^ found an increase in *Batrachochytrium dendrobatidis* positive samples in the southwestern Amazonia, coinciding with reported amphibian declines in neighboring high elevation sites on Andean slopes of Peru. Considering that the pathogen thrives in cool, moist environments in high-elevation tropical rainforests, our results indicate that anuran species occurring in BR4 would be the most susceptible to *Batrachochytrium dendrobatidis* expansion and anuran species populations in this region should be careful monitored.

Currently, the integrity of the Amazonia is under pressure from dam constructions, deforestation, climate change and unsustainable economic activities^12,56,57^. For example, large dam constructions could not only block movements that connect anuran populations, but also result in the loss of terrestrial habitats by flooding indigenous lands and conservation units that are protecting several endemic and undescribed species^56,58^. Based on the predictions of Latrubesse *et al*.^57^, if the planned dams are constructed in Amazon basin, BR4 and BR5 will be the most impacted biogeographic regions. These regions harbor the highest anuran species richness, with most species showing a restricted range-size distribution. Furthermore, future projections indicated that agricultural expansion and climate variability will change regional precipitation patterns in Amazonia^11,59,60^. Sorribas *et al*.^60^ projected a decrease in river discharges for eastern basins, and decrease in inundation in central and lower Amazonia. These projections are worrisome because most of these changes will occur with replacement of tropical forest by seasonal forest and tropical savanna^59^. The likelihood of “savannization” of parts of Amazonia could favor the invasion of these altered areas by anuran species from the Cerrado that are more resistant to desiccation and have more generalized reproductive mode^61^. Taken together, these actions could threat the integrity of the ecosystem, and alter the patterns of species distribution.

## Methods

### Species distribution data

We downloaded range maps for all species of anurans recorded in the Amazonia region from the IUCN *version* 2015.2^62^. Then, we overlaid the range maps into grid cells at 50 × 50 km to generate a presence–absence matrix and determine the number of species by grid cell. We considered the extent of the Amazonia region based on the Cracraft^14^ delimitation and subsequently modified by Silva *et al*.^15^. We excluded all species from other biomes (e.g. Cerrado) with marginal occurrences inside the Amazonia region. In the end, a total of 577 anuran species were considered for the regionalization process (see Appendix S1 in Supporting Information). We standardized the nomenclature of anuran species following the *Amphibian Species of the World* (Frost)^63^.

We are aware that biogeographical inferences are affected by incomplete taxonomic and distributional knowledge^7,64^. Although the IUCN anuran maps might include either over- or underpredictions mainly in megadiverse tropical regions^49^, range maps have been used to investigate amphibian regionalization across a range of spatial scales^4,6,8^. Furthermore, from a macroecological perspective, range maps have performed very well at resolutions greater than 50 × 50 km^65^. However, to understand the effects of anuran species that were described recently or whose range size distribution is underpredicted, we also analyzed three other datasets excluding from the presence–absence matrix the small-ranged species that occurred in only one (501 species remained in the matrix), two (440 species) and three (418 species) grid cells. Biogeographical regions delimited using the 577 anuran species and the three datasets excluding small-ranged species were similar. Therefore, we will present only the results considering the 577 anuran species (see Appendix S2 in Supporting Information for a discussion about the results).

### Clustering procedures

We used the *recluster*.*region* algorithm^66,67^ available in the *recluster* R package^68^ to identify the biogeographic regions in Amazonia with distinct anuran species compositions. This algorithm calculates the dissimilarity of species compositions between each pair of grid cells using the Simpson index (β_sim_), which is not affected by variations in species richness:

$${\rm{\beta }}\mathrm{sim}=1-\frac{{\rm{\min }}(b,c)}{a+\,\min (b,c)},$$

where component *a* comprises the total number of species shared by two grids; component *b* comprises the total number of species that occur in the neighboring grids but not in the focal one; and component *c* comprises the total number of species that occur in the focal grid but not in the neighboring one. This index is a desirable choice for regionalization because species replacement is largely influenced by vicariance and endemism phenomena^7^. Then, we used Ward hierarchical clustering to convert dissimilarity matrices into bifurcated dendrograms^69^. This method performs better in a simulation for recognizing regionalization patterns than other hierarchical clustering methods commonly used for biogeographical analyses^67^. According to Dapporto *et al*.^66^, due to a high frequency of ties and zero values produced by beta-diversity turnover indices, the topology and bootstrap support of dendrograms are affected by the order of areas in the original presence–absence matrix. To avoid these problems, the *recluster*.*region* algorithm produces *n* trees (*n* = 50 by default) by randomly reordering the areas in the original dissimilarity matrix. Next, the function cuts these trees at different k_1_ − k_n_ levels (i.e. the number of regions to be identified), producing *n* matrices of areas x cluster membership^67^. We delimited the maximum number of regions at 50 clusters. Lastly, to identify the number of regions, the function provides the explained dissimilarity^2^ and the mean silhouette width^70^ for all the clustering solutions. The explained dissimilarity is represented by the ratio between the sums of the mean dissimilarities among members of different clusters and the sum of all dissimilarities in the matrix. This method maximizes the between-cluster variation relative to the within-cluster variation. According to Holt *et al*.^2^, clusters that reach the threshold value of 90% are an appropriate choice for establishing a suitable tree cut. The mean silhouette width measures the strength of any of the partitions of objects from a dissimilarity matrix. This index ranges between −1 and +1, with negative values indicating that cells are probably located in incorrect clusters^70^. Here, we identify biogeographic regions based on the number of clusters that considerably improved the explained dissimilarity and the mean silhouette width together. For that, we first found the number of cluster that reach the threshold value of 90% proposed by Holt *et al*.,^2^, then we delimited the cluster number when the mean silhouette value stopped increasing.

### Predictor variables

To test the potential correlates in the anuran cluster patterns, we obtained current and historical climate data, topographic data, riverine barriers and vegetation structure, which are detailed below:

*Current climate variables* – the selected climate variables were: i) average annual maximum temperature (AMAXTE); ii) average annual minimum temperature (AMINTE); iii) temperature seasonality (TESE); iv) annual precipitation (APRE); v) precipitation range (PRER); and vi) precipitation seasonality (PRSE). These variables were chosen because they describe a central tendency as well as the variation in the descriptors representing physiological limits or dispersal barriers for anurans^6,8,25^. These data were downloaded from the WorldClim database at a resolution of 5′ arc-minutes^71^.

*Pleistocene climate variables* – we downloaded the values of annual precipitation and annual mean temperature from three models of the Last Glacial Maximum (LGM; CCSM4, MIROC-ESM, MPI-ESM-P) available from the WorldClim database (http://www.worldclim.org/downscaling). Following Moura *et al*.^10^ we calculated two historical difference in climate variables: i) historical difference in annual precipitation (HDP) was calculated by the difference between current and LGM annual precipitation; and ii) historical difference in annual mean temperature (HDT) was calculated by the difference between current and LGM annual mean temperature. These two measures indicate the historical variation in water availability and energy input respectively. In order to couple with the variations among the circulation models, we averaged the grid cell values among them prior to the calculation of historical difference^10^.

*Topographic variables* – for each grid cell, we calculated six measures of topographic heterogeneity based on elevation data (~1 × 1 km resolution) available at https://lta.cr.usgs.gov/GTOPO30. These measures were: i) maximum elevation (TOPOMAX); ii) minimum elevation (TOPOMIN); iii) elevational standard deviation (TOPOSTD); iv) slope range (SLOPERAN); v) slope standard deviation (SLOPESTD); and vi) aspect standard deviation (ASPECTSTD).

**Figure 4:**
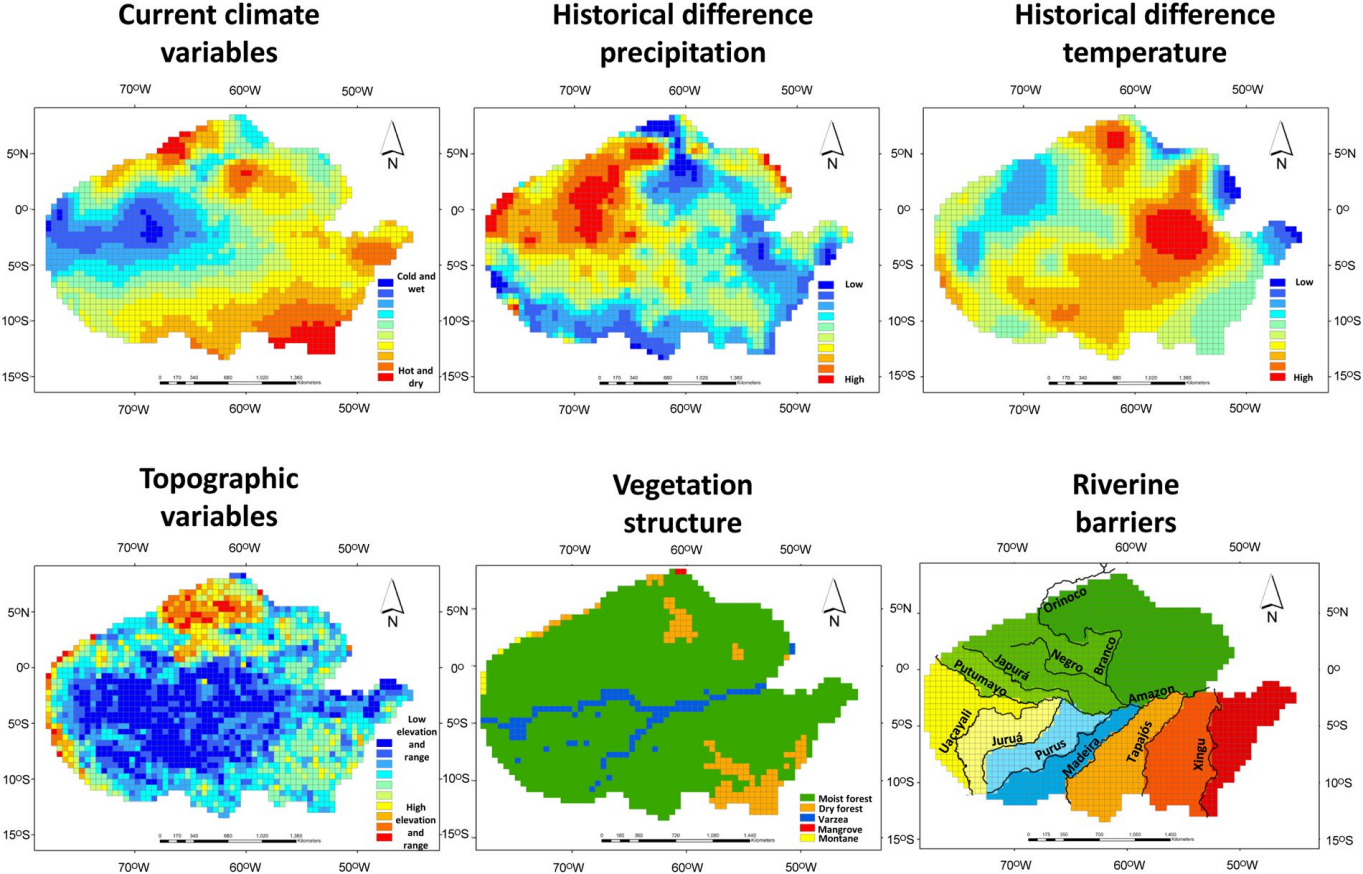
Distribution of predictor variables used to evaluate the anuran biogeographical regions in the Amazonia. *Current climate variables* - first axis of principal components analyses (PCA) with precipitation and temperature variables (AMAXTE, AMINTE, TESE, APRE, PRER and PRSE); *Historical difference precipitation (HDP)* - difference between current and Last Glacial Maximum (LGM) annual precipitation; *Historical difference temperature (HDT)* - difference between current and LGM annual mean temperature; *Topographic variables* - first axis of PCA with elevation and slope variables (TOPOMAX, TOPOMIN, TOPOSTD, SLOPERAN, SLOPESTD and ASPECTSTD); *Vegetation structure* – Amazonia ecoregions based on the classification of Olson *et al*.^9^; and *Riverine barriers* - classification of grids based on the banks of ten major rivers in Amazonia. Maps generated using ESRI ArcMap 9.2. https://www.esri.com/.

*Riverine barrier* – we categorized the grid cells into different regions based on the banks of the largest rivers in the Amazonia in terms of water discharge^72^ and preview studies^14,17^: i) Amazon (mean annual discharge − 209000 m^3^/s), ii) Orinoco (35000 m^3^/s), iii) Madeira (32000 m^3^/s), iv) Negro (28400 m^3^/s), v) Japurá (18600 m^3^/s), vi) Tapajós (13500 m^3^/s), vii) Purus (11000 m^3^/s), viii) Xingu (9700 m^3^/s), ix) Uacayali (9544 m^3^/s), x) Putumayo (8760 m^3^/s), xi)Tocantins (8440 m^3^/s) and xii) Rio Branco (1462 m^3^/s) (Fig. 4). These data were downloaded from the database of USGS at https://www.sciencebase.gov/catalog/item/56814fc2e4b0a04ef492213e.

*Vegetation structure* – we used the classification of Olson *et al*.^9^ to determine the percentage of vegetation type covering each grid (Fig. 4). The main vegetation types observed were moist forest, dry forest, varzea, mangrove and montane.

### Statistical analysis

#### Correlates of biogeographical regions

To reduce the dimensionality and number of correlations between variables in our database, we performed three separate principal components analyses (PCA), a first one with the set of current climate variables (AMAXTE, AMINTE, TESE, APRE, PRER and PRSE), a second one with the set of topographic variables (TOPOMAX, TOPOMIN, TOPOSTD, SLOPERAN, SLOPESTD and ASPECTSTD) and a final one with the percentage of each vegetation type. Therefore, for the subsequent analysis, we used nine variables: i) the first two axes from the current climate variables (CURE.PC1 and CURE.PC2), ii) the first two axes from the topographic variables (TOPO.PC1 and TOPO.PC2), iii) the first two axes from the vegetation structure (VEGE.PC1 and VEGE.PC2), iv) two Pleistocene climate variations (HDP and HDT), and v) the classification of grids based on the banks of eight major rivers. We also evaluated the correlation between original environmental variables and the first two axes of the three PCAs using significance tests of Pearson correlation coefficients (see Appendix S3 in Supporting Information).

We used multinomial logistic regression models to investigate the influence of predictor variables in explaining the anuran biogeographic regions^8,10^. To determine the optimal model related to biogeographical regions, we started with a full model containing all explanatory variables. Then we generated sub-model sets from the full model using the dredge function implemented in the MuMIn package^73^. We used Akaike’s information criterion corrected for small sample sizes (AICc^74^) to determine the optimal model. The AICc is calculated for each model from its log-likelihood and the number of parameters, and the model with the lowest AICc is judged to be the best of the candidate models^74^. Furthermore, to evaluate model selection uncertainty, we used Akaike weights (ὠ), which express the likelihood of each model given the data and the set of candidate models. Finally, we used variation partitioning analysis^75^ to partition the total percentage of variation into unique contributions of the sets of predictors of the best model.

All analyses were performed with R 3.2.3 software^76^.

### Data accessibility statement

All data were gathered on public databases that are available on-line.

## Electronic supplementary material

Supplementary information
