# Supplementary material for: The influence of riverine barriers, climate, and topography on the biogeographic regionalization of Amazonian anurans
Source: Sci Rep. 2018 Feb 21;8:3427. doi: 10.1038/s41598-018-21879-9 (PMC5821848; doi:10.1038/s41598-018-21879-9)
Supplement: Supplementary file 1 — Supplementary information [file 41598_2018_21879_MOESM1_ESM.pdf]

SUPPLEMENTARY INFORMATION

The influence of riverine barriers, climate and topography on the biogeographic regionalization of Amazonian anurans

Marcela Brasil de Castro Godinho and Fernando Rodrigues da Silva

Scientific Reports

APPENDIX S1 – List of anuran species to Amazonia download from IUCN *version* 2015.2 and the number of grid cells that each species occurred.

Anuran species	Nº of grid cells	Anuran species	Nº of grid cells
Adelastes hylonomos	1	*Leptodactylus validus*	215
Adelophryne adiastola	98	*Leptodactylus vastus*	30
Adelophryne gutturosa	121	*Leptodactylus wagneri*	100
Adelophryne patamona	3	*Lithobates catesbeianus*	6
Adelphobates castaneoticus	24	*Lithobates palmipes*	1862
Adelphobates galactonotus	180	*Metaphryniscus sosai*	2
Adelphobates quinquevittatus	493	*Minyobates steyermarki*	1
Adenomera andreae	1722	*Myersiohyla aromatica*	1
Adenomera heyeri	83	*Myersiohyla inparquesi*	2
Adenomera hylaedactyla	1859	*Myersiohyla kanaima*	3
Adenomera lutzi	9	*Myersiohyla loveridgei*	2
Adenomera martinezi	90	*Noblella lochites*	4
Agalychnis buckleyi	28	*Noblella myrmecoides*	179
Agalychnis hulli	22	*Nyctimantis rugiceps*	22
Allobates brunneus	624	*Nymphargus chancas*	1
Allobates caeruleodactylus	4	*Nymphargus cochranae*	4
Allobates conspicuus	152	*Nymphargus laurae*	1
Allobates crombiei	12	*Nymphargus mariae*	3
Allobates femoralis	1679	*Nymphargus megacheirus*	2
Allobates fuscellus	254	*Nymphargus posadae*	8
Allobates gasconi	49	*Nymphargus siren*	2
Allobates granti	21	*Nymphargus wileyi*	1
Allobates insperatus	13	*Oreobates cruralis*	19
Allobates kingsburyi	4	*Oreobates quixensis*	509
Allobates marchesianus	1160	*Oreobates saxatilis*	5
Allobates masniger	8	*Oreophrynella cryptica*	2
Allobates melanolaemus	6	*Oreophrynella dendronastes*	1
Allobates myersi	36	*Oreophrynella huberi*	4
Allobates nidicola	1	*Oreophrynella macconnelli*	1

Allobates ornatus	2	*Oreophrynella nigra*	2
Allobates paleovarzensis	8	*Oreophrynella quelchii*	1
Allobates subfolionidificans	2	*Oreophrynella vasquezi*	3
Allobates sumtuosus	84	*Oreophrynella weiassipuensis*	1
Allobates trilineatus	222	*Osornophryne bufoniformis*	3
Allobates undulatus	2	*Osornophryne guacamayo*	1
Allobates vanzolinius	46	*Osornophryne sumacoensis*	1
Allobates zaparo	24	*Osteocephalus alboguttatus*	10
Allophryne ruthveni	1010	*Osteocephalus buckleyi*	1390
Amazophrynella bokermanni	18	*Osteocephalus cabrerai*	566
Amazophrynella minuta	1637	*Osteocephalus castaneicola*	11
Ameerega bassleri	14	*Osteocephalus deridens*	29
Ameerega bilinguis	18	*Osteocephalus fuscifacies*	8
Ameerega cainarachi	1	*Osteocephalus heyeri*	11
Ameerega flavopicta	73	*Osteocephalus leoniae*	14
Ameerega hahneli	1309	*Osteocephalus leprieurii*	1204
Ameerega ingeri	1	*Osteocephalus mimeticus*	63
Ameerega macero	30	*Osteocephalus mutabor*	25
Ameerega parvula	79	*Osteocephalus oophagus*	349
Ameerega petersi	52	*Osteocephalus planiceps*	247
Ameerega picta	502	*Osteocephalus subtilis*	60
Ameerega pongoensis	9	*Osteocephalus taurinus*	1730
Ameerega pulchripecta	8	*Osteocephalus verruciger*	13
Ameerega rubriventris	7	*Osteocephalus yasuni*	61
Ameerega silverstonei	2	*Otophryne pyburni*	171
Ameerega trivittata	1456	*Otophryne robusta*	15
Anomaloglossus ayarzaguenai	4	*Otophryne steyermarki*	17
Anomaloglossus baeobatrachus	243	*Phyllomedusa atelopoides*	168
Anomaloglossus beebei	1	*Phyllomedusa azurea*	4
Anomaloglossus breweri	1	*Phyllomedusa baltea*	2
Anomaloglossus degranvillei	71	*Phyllomedusa bicolor*	1571
Anomaloglossus guanayensis	2	*Phyllomedusa boliviana*	111
Anomaloglossus kaiei	10	*Phyllomedusa camba*	200
Anomaloglossus murisipanensis	2	*Phyllomedusa coelestis*	35
Anomaloglossus parimae	1	*Phyllomedusa hypochondrialis*	1182
Anomaloglossus parkerae	4	*Phyllomedusa palliata*	301
Anomaloglossus praderioi	1	*Phyllomedusa tarsius*	944
Anomaloglossus roraima	1	*Phyllomedusa tomopterna*	1662
Anomaloglossus rufulus	2	*Phyllomedusa vaillantii*	1490
Anomaloglossus shrevei	10	*Physalaemus albonotatus*	1
Anomaloglossus stepheni	13	*Physalaemus centralis*	5

Anomaloglossus tamacuarensis	2	*Physalaemus cuvieri*	716
Anomaloglossus tepuyensis	4	*Physalaemus ephippifer*	668
Anomaloglossus triunfo	1	*Physalaemus fischeri*	40
Anomaloglossus wothuja	1	*Physalaemus nattereri*	36
Aparasphenodon venezolanus	46	*Phyzelaphryne miriamae*	434
Atelopus andinus	4	*Pipa arrabali*	396
Atelopus epikeisthos	1	*Pipa aspera*	36
Atelopus flavescens	1	*Pipa pipa*	1665
Atelopus franciscus	11	*Pipa snethlageae*	675
Atelopus palmatus	1	*Pleurodema brachyops*	125
Atelopus planispina	5	*Pristimantis aaptus*	38
Atelopus pulcher	10	*Pristimantis acuminatus*	244
Atelopus pyrodactylus	1	*Pristimantis altamazonicus*	531
Atelopus seminiferus	4	*Pristimantis altamnis*	6
Atelopus siranus	2	*Pristimantis ardalonychus*	7
Atelopus spumarius	349	*Pristimantis aureolineatus*	40
"Centrolene" azulae	1	*Pristimantis aureoventris*	1
Centrolene bacatum	2	*Pristimantis avius*	1
Centrolene buckleyi	2	*Pristimantis bearsei*	4
Centrolene hybrida	1	*Pristimantis buccinator*	18
Centrolene lemniscatum	1	*Pristimantis buckleyi*	2
"Centrolene" medemi	1	*Pristimantis cantitans*	4
Ceratophrys cornuta	1562	*Pristimantis carvalhoi*	397
Ceuthomantis aracamuni	2	*Pristimantis chiastonotus*	115
Ceuthomantis cavernibardus	1	*Pristimantis chloronotus*	3
Ceuthomantis duellmani	2	*Pristimantis citriogaster*	2
Chiasmocleis albopunctata	78	*Pristimantis colonensis*	1
Chiasmocleis anatipes	43	*Pristimantis conspicillatus*	269
Chiasmocleis antenori	132	*Pristimantis corrugatus*	1
Chiasmocleis avilapiresae	397	*Pristimantis croceoinguinis*	116
Chiasmocleis bassleri	606	*Pristimantis delius*	1
Chiasmocleis carvalhoi	56	*Pristimantis dendrobatoides*	4
Chiasmocleis devriesi	1	*Pristimantis devillei*	1
Chiasmocleis hudsoni	372	*Pristimantis diadematus*	170
Chiasmocleis magnova	3	*Pristimantis eriphus*	2
Chiasmocleis shudikarensis	990	*Pristimantis ernesti*	1
Chiasmocleis tridactyla	104	*Pristimantis eurydactylus*	137
Chiasmocleis ventrimaculata	214	*Pristimantis exoristus*	3
Chimerella mariaelenae	4	*Pristimantis fenestratus*	1027
"Cochranella" duidaeana	3	*Pristimantis frater*	2
"Cochranella" geijskesi	2	*Pristimantis galdi*	7
Cochranella resplendens	48	*Pristimantis gutturalis*	76

"Cochranella" riveroi	2	*Pristimantis imitatrix*	20
Colostethus argyrogaster	10	*Pristimantis incanus*	2
Colostethus fugax	5	*Pristimantis incomptus*	1
Cruziohyla craspedopus	624	*Pristimantis infraguttatus*	1
Ctenophryne carpish	3	*Pristimantis inguinalis*	65
Ctenophryne geayi	1767	*Pristimantis inusitatus*	5
Dendrobates leucomelas	280	*Pristimantis jester*	4
Dendrobates nubeculosus	1	*Pristimantis kichwarum*	16
Dendrobates tinctorius	201	*Pristimantis lacrimosus*	141
Dendropsophus acreanus	252	*Pristimantis lanthanites*	226
Dendropsophus anataliasiasi	95	*Pristimantis leoni*	2
Dendropsophus aperomeus	4	*Pristimantis librarius*	3
Dendropsophus bifurcus	368	*Pristimantis lirellus*	4
Dendropsophus bokermanni	381	*Pristimantis luscombei*	14
Dendropsophus branneri	16	*Pristimantis lythrodes*	7
Dendropsophus brevifrons	583	*Pristimantis malkini*	304
Dendropsophus cachimbo	1	*Pristimantis marahuaka*	1
Dendropsophus delarivai	9	*Pristimantis marmoratus*	320
Dendropsophus gaucheri	5	*Pristimantis martiae*	444
Dendropsophus haraldschultzi	308	*Pristimantis melanogaster*	2
Dendropsophus joannae	2	*Pristimantis memorans*	1
Dendropsophus juliani	4	*Pristimantis mendax*	28
Dendropsophus koechlini	352	*Pristimantis muscosus*	2
Dendropsophus leali	575	*Pristimantis nephophilus*	4
Dendropsophus leucophyllatus	1627	*Pristimantis nigrogriseus*	4
Dendropsophus marmoratus	1494	*Pristimantis ockendeni*	300
Dendropsophus mathiassoni	8	*Pristimantis orcus*	80
Dendropsophus melanargyreus	504	*Pristimantis orphnolaimus*	14
Dendropsophus microcephalus	1395	*Pristimantis paululus*	24
Dendropsophus minimus	1	*Pristimantis percnopterus*	3
Dendropsophus minusculus	79	*Pristimantis peruvianus*	446
Dendropsophus minutus	1795	*Pristimantis petersi*	6
Dendropsophus miyatai	489	*Pristimantis prolatus*	4
Dendropsophus nanus	959	*Pristimantis pruinatus*	2
Dendropsophus parviceps	1384	*Pristimantis pseudoacuminatus*	22
Dendropsophus pauiniensis	125	*Pristimantis pugnax*	3
Dendropsophus reichlei	7	*Pristimantis pulvinatus*	102
Dendropsophus rhodopeplus	435	*Pristimantis quaquaversus*	38
Dendropsophus riveroi	721	*Pristimantis rhabdolaemus*	4
Dendropsophus rossalleni	495	*Pristimantis rhodostichus*	1
Dendropsophus rubicundulus	194	*Pristimantis rubicundus*	4
Dendropsophus sarayacuensis	609	*Pristimantis rufioculis*	2

Dendropsophus schubarti	135	*Pristimantis saltissimus*	8
Dendropsophus soaresi	3	*Pristimantis savagei*	2
Dendropsophus timbeba	68	*Pristimantis schultei*	1
Dendropsophus tintinnabulum	20	*Pristimantis skydmainos*	66
Dendropsophus triangulum	867	*Pristimantis stictoboubonus*	2
Dendropsophus walfordi	450	*Pristimantis tamsitti*	3
Dendropsophus xapuriensis	60	*Pristimantis toftae*	44
Dermatonotus muelleri	58	*Pristimantis trachyblepharis*	2
Dischidodactylus colonnelloi	2	*Pristimantis unistrigatus*	1
Dischidodactylus duidensis	1	*Pristimantis variabilis*	295
Dryaderces inframaculata	1	*Pristimantis ventrimarmoratus*	135
Dryaderces pearsoni	28	*Pristimantis vilarsi*	579
Ecnomiohyla tuberculosa	135	*Pristimantis wagteri*	2
Edalorhina nasuta	3	*Pristimantis waoranii*	2
Edalorhina perezi	454	*Pristimantis w-nigrum*	7
Elachistocleis carvalhoi	9	*Pristimantis yaviensis*	2
Elachistocleis helianneae	385	*Pristimantis zeuctotylus*	274
Elachistocleis ovalis	1684	*Pristimantis zimmermanae*	24
Elachistocleis surinamensis	19	*Pristimantis zoilae*	3
Elachistocleis surumu	3	*Proceratophrys concavitympanum*	1
Eleutherodactylus johnstonei	2	*Pseudis boliviana*	319
Engystomops freibergi	652	*Pseudis caraya*	263
Engystomops petersi	148	*Pseudis laevis*	24
Engystomops pustulosus	25	*Pseudis paradoxa*	1518
Espadarana audax	1	*Pseudopaludicola boliviana*	875
Espadarana durrellorum	8	*Pseudopaludicola canga*	10
Excidobates captivus	2	*Pseudopaludicola ceratophryes*	182
Gastrotheca andaquiensis	9	*Pseudopaludicola llanera*	33
Gastrotheca longipes	32	*Ranitomeya amazonica*	9
Gastrotheca monticola	1	*Ranitomeya benedicta*	9
Gastrotheca nicefori	2	*Ranitomeya fantastica*	6
Gastrotheca testudinea	39	*Ranitomeya flavovittata*	9
Gastrotheca weinlandii	13	*Ranitomeya imitator*	9
Hamptophryne boliviana	1617	*Ranitomeya reticulata*	60
Hemiphractus bubalus	32	*Ranitomeya sirensis*	34
Hemiphractus helioi	50	*Ranitomeya summersi*	1
Hemiphractus johnsoni	11	*Ranitomeya uakarii*	119
Hemiphractus proboscideus	138	*Ranitomeya vanzolinii*	55
Hemiphractus scutatus	423	*Ranitomeya variabilis*	4
Hyalinobatrachium cappellei	79	*Ranitomeya ventrimaculata*	871
Hyalinobatrachium fleischmanni	1	*Rhaebo glaberrimus*	196

Hyalinobatrachium iaspidiense	28	*Rhaebo guttatus*	1834
Hyalinobatrachium pellucidum	1	*Rhaebo nasicus*	34
Hyalinobatrachium ruedai	9	*Rhinella acutirostris*	151
Hyalinobatrachium taylori	192	*Rhinella arborescandens*	1
Hydrolaetare dantasi	66	*Rhinella castaneotica*	997
Hydrolaetare schmidti	884	*Rhinella ceratophrys*	354
"Hyla" imitator	1	*Rhinella cristinae*	1
Hyloscirtus albopunctulatus	30	*Rhinella dapsilis*	93
Hyloscirtus lindae	5	*Rhinella festae*	13
Hyloscirtus phyllognathus	18	*Rhinella granulosa*	1416
Hyloscirtus psarolaimus	1	*Rhinella humboldti*	28
Hyloscirtus staufferorum	1	*Rhinella lescurei*	14
Hyloscirtus torrenticola	6	*Rhinella magnussoni*	4
Hyloxalus azureiventris	3	*Rhinella margaritifera*	1876
Hyloxalus bocagei	24	*Rhinella marina*	1876
Hyloxalus cevallosi	3	*Rhinella martyi*	103
Hyloxalus chlorocraspedus	1	*Rhinella nesiotes*	2
Hyloxalus craspedoceps	1	*Rhinella ocellata*	117
Hyloxalus eleutherodactylus	1	*Rhinella poeppigii*	38
Hyloxalus faciopunctulatus	4	*Rhinella proboscidea*	176
Hyloxalus fuliginosus	1	*Rhinella roqueana*	176
Hyloxalus idiomelus	2	*Rhinella rubescens*	37
Hyloxalus insulatus	1	*Rhinella schneideri*	177
Hyloxalus maculosus	2	*Rulyrana flavopunctata*	18
Hyloxalus mittermeieri	1	*Rulyrana saxiscandens*	2
Hyloxalus nexipus	23	*Scarthyla goinorum*	515
Hyloxalus patitae	1	*Scinax baumgardneri*	3
Hyloxalus peruvianus	200	*Scinax blairi*	17
Hyloxalus pulchellus	4	*Scinax boesemani*	1413
Hyloxalus sauli	19	*Scinax chiquitanus*	43
Hyloxalus shuar	6	*Scinax cruentommus*	858
Hyloxalus sordidatus	6	*Scinax danae*	4
Hypodactylus dolops	4	*Scinax eurydice*	8
Hypodactylus elassodiscus	2	*Scinax exiguus*	9
Hypodactylus nigrovittatus	309	*Scinax funereus*	284
Hypsiboas albopunctatus	203	*Scinax fuscomarginatus*	254
Hypsiboas boans	1827	*Scinax fuscovarius*	181
Hypsiboas calcaratus	1440	*Scinax garbei*	1154
Hypsiboas cinerascens	1793	*Scinax ictericus*	30
Hypsiboas crepitans	245	*Scinax iquitorum*	25
Hypsiboas dentei	58	*Scinax karenanneae*	4
Hypsiboas fasciatus	1526	*Scinax kennedyi*	6

Hypsiboas fuentei	1	*Scinax lindsayi*	51
Hypsiboas geographicus	1876	*Scinax nebulosus*	1236
Hypsiboas hobbsi	86	*Scinax oreites*	11
Hypsiboas hutchinsi	39	*Scinax pedromedinae*	52
Hypsiboas jimenezi	2	*Scinax proboscideus*	71
Hypsiboas lanciformis	1351	*Scinax rostratus*	115
Hypsiboas lemai	20	*Scinax ruber*	1832
Hypsiboas leucocheilus	3	*Scinax wandae*	31
Hypsiboas liliae	7	*Scinax x-signatus*	1714
Hypsiboas microderma	127	*Sphaenorhynchus carneus*	559
Hypsiboas multifasciatus	486	*Sphaenorhynchus dorisae*	508
Hypsiboas nympha	88	*Sphaenorhynchus lacteus*	1589
Hypsiboas ornatissimus	136	*Stefania ackawaio*	4
Hypsiboas pulidoi	2	*Stefania ayangannae*	4
Hypsiboas punctatus	1875	*Stefania breweri*	1
Hypsiboas raniceps	1275	*Stefania coxi*	4
Hypsiboas rhythmicus	4	*Stefania evansi*	24
Hypsiboas roraima	2	*Stefania ginesi*	4
Hypsiboas sibleszi	68	*Stefania goini*	2
Hypsiboas tepuianus	36	*Stefania marahuaquensis*	2
Hypsiboas wavrini	838	*Stefania oculosa*	4
Leptodactylus bolivianus	1629	*Stefania percristata*	4
Leptodactylus chaquensis	24	*Stefania riae*	2
Leptodactylus colombiensis	4	*Stefania riveroi*	2
Leptodactylus didymus	7	*Stefania roraimae*	6
Leptodactylus diedrus	269	*Stefania satelles*	6
Leptodactylus discodactylus	412	*Stefania scalae*	14
Leptodactylus elenae	88	*Stefania schuberti*	2
Leptodactylus fragilis	6	*Stefania tamacuarina*	1
Leptodactylus furnarius	9	*Stefania woodleyi*	5
Leptodactylus fuscus	1550	*Strabomantis cornutus*	12
Leptodactylus knudseni	1567	*Strabomantis sulcatus*	393
Leptodactylus labyrinthicus	106	*Synapturanus mirandaribeiroi*	488
Leptodactylus latrans	1227	*Synapturanus rabus*	69
Leptodactylus leptodactyloides	1438	*Synapturanus salseri*	166
Leptodactylus lineatus	1576	*Tepuihyla aecii*	1
Leptodactylus lithonaetes	56	*Tepuihyla edelcae*	9
Leptodactylus longirostris	276	*Tepuihyla exophthalma*	2
Leptodactylus myersi	103	*Tepuihyla luteolabris*	2
Leptodactylus mystaceus	1768	*Tepuihyla rodriguezi*	8
Leptodactylus mystacinus	2	*Tepuihyla warreni*	2
Leptodactylus paraensis	213	*Teratohyla amelie*	1
Leptodactylus pentadactylus	1401	*Teratohyla midas*	501

Leptodactylus petersii	1645	*Trachycephalus coriaceus*	727
Leptodactylus podicipinus	333	*Trachycephalus hadroceps*	91
Leptodactylus pustulatus	177	*Trachycephalus resinifictrix*	1465
Leptodactylus rhodomystax	1602	*Trachycephalus typhonius*	1874
Leptodactylus rhodonotus	111	*Vitreorana gorzulae*	5
Leptodactylus riveroi	324	*Vitreorana helenae*	1
Leptodactylus rugosus	67	*Vitreorana ritae*	7
Leptodactylus sabanensis	8		
Leptodactylus stenodema	1450		
Leptodactylus syphax	20		

APPENDIX S2 – Determining biogeographical regions considering different presence-absence matrix.

To understand the effects of anuran species that were described recently or whose range size distribution is underpredicted, we delimeted biogeographical regions using four different datasets: i) all anuran species downloaded from IUCN - containing 577 species; ii) excluding small-ranged species that occurred in only one grid cell - containing 501 species; iii) excluding small-ranged species that occurred in only two grid cells - containing 440 species; and iv) excluding small-ranged species that occurred in only three grid cells - containing 418 species. Independently of the matrix analyzed, we found seven biogeographical regions with similar distributions (Figure S2, Table S2).

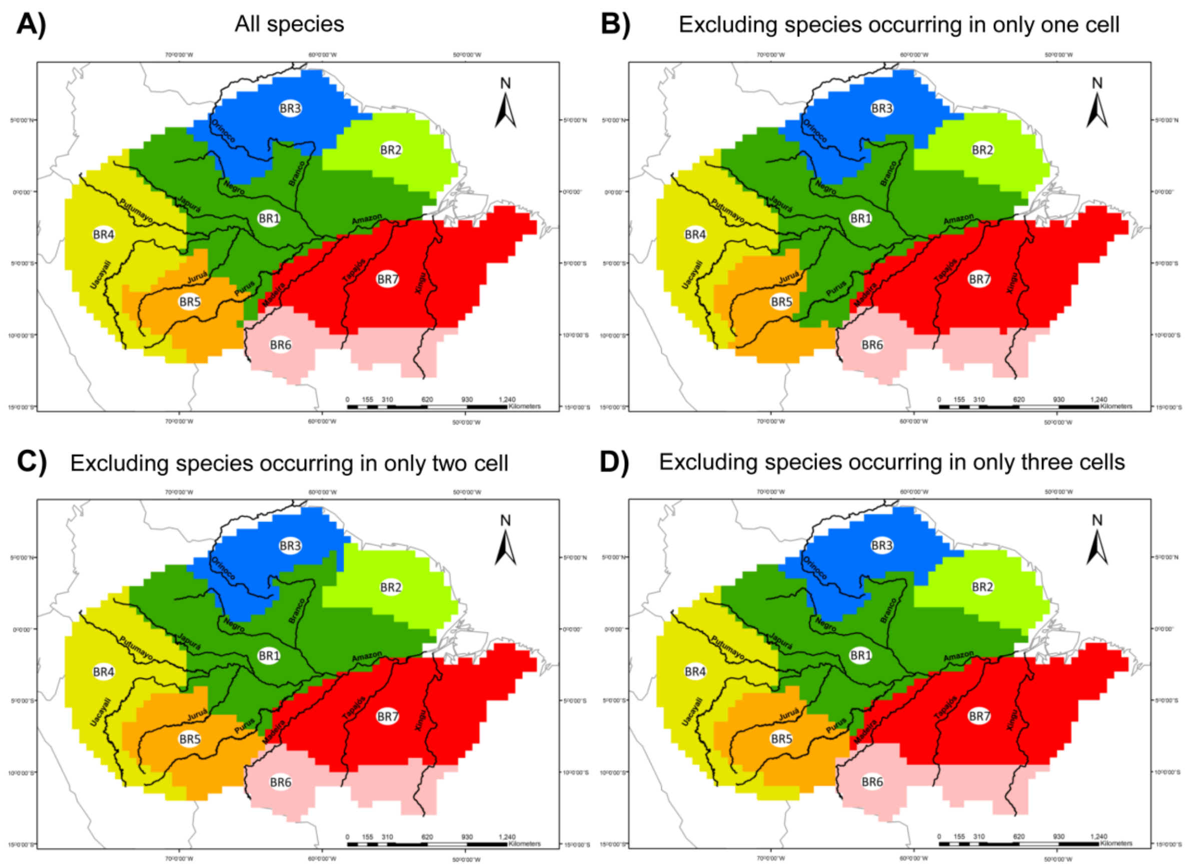

Figure S2. Regionalization of anuran dissimilarity into seven biogeographical regions (BR) in the Amazonia considering different datasets. A) all anuran species downloaded from IUCN - containing 577 species ; B) dataset excluding small-ranged species that occurred in only one grid cell - containing 501 species; C) dataset excluding small-ranged species that occurred in only two grid cells - containing 440 species; and D) dataset excluding small-ranged species that occurred in only three grid cells - containing 418 species. Black lines represent the ten major rivers in Amazonia. Map generated using ESRI ArcMap 9.2. https://www.esri.com

TABLE S2. Values of the mean silhouette width (Silh) and the explained dissimilarity (ex.diss) for delimitation of biogeographical regions using four different datasets: i) all anuran species downloaded from IUCN - containing 577 species; ii) excluding small-ranged species that occurred in only one grid cell - containing 501 species; iii) excluding small-ranged species that occurred in only two grid cells - containing 440 species; and iv) excluding small-ranged species that occurred in only three grid cells -containing 418 species. The mean silhouette width measures the strength of any of the partitions of objects from a dissimilarity matrix.The explained dissimilarity maximizes between-cluster variation relative to within-cluster variation. In bold the number of clusters selected.

Nº of clusters	All anuran species		Excluding anuran species in only 1 grid		Excluding anuran species in only 2 grids		Excluding anuran species in only 3 grids	
	Silh	ex.diss	Silh	ex.diss	Silh	ex.diss	Silh	ex.diss
2	0.410	0.384	0.407	0.392	0.384	0.474	0.391	0.458
3	0.291	0.707	0.292	0.712	0.281	0.691	0.315	0.745
4	0.314	0.841	0.316	0.839	0.314	0.833	0.316	0.839
5	0.298	0.876	0.292	0.879	0.306	0.877	0.309	0.869
6	0.313	0.909	0.307	0.909	0.319	0.907	0.312	0.906
**7**	**0.336**	**0.925**	**0.335**	**0.924**	**0.333**	**0.919**	**0.332**	**0.906**
8	0.334	0.937	0.323	0.938	0.328	0.938	0.330	0.938
9	0.346	0.942	0.304	0.943	0.346	0.943	0.332	0.944
10	0.321	0.953	0.331	0.952	0.314	0.953	0.307	0.952
11	0.319	0.961	0.301	0.962	0.308	0.963	0.302	0.962
12	0.321	0.966	0.301	0.967	0.307	0.968	0.301	0.968
13	0.335	0.968	0.314	0.971	0.321	0.971	0.314	0.970
14	0.355	0.971	0.335	0.973	0.324	0.972	0.329	0.973
15	0.341	0.975	0.348	0.976	0.342	0.975	0.351	0.976
16	0.341	0.979	0.341	0.978	0.336	0.978	0.360	0.978
17	0.344	0.981	0.355	0.981	0.344	0.981	0.368	0.980
18	0.348	0.981	0.345	0.982	0.356	0.981	0.370	0.981
19	0.337	0.983	0.359	0.983	0.357	0.983	0.361	0.982
20	0.354	0.984	0.351	0.984	0.345	0.984	0.346	0.984

APPENDIX S3.

Table S3. Pearson correlations (r) between environmental variables and the two axes of principal components analyses (PCA) used in the manuscript. The highest correlation values for each axis are in bold.

	FIRST AXIS PCA		SECOND AXIS PCA	
	r	p	r	p
**CONTEMPORARY CLIMATE**				
Average annual maximum temperature	0.39	<0.001	-0.52	<0.001
Average annual minimum temperature	-0.61	<0.001	**-0.88**	**<0.001**
Temperature seasonality	0.81	<0.001	-0.15	<0.001
Annual precipitation	**0.95**	**<0.001**	-0.04	0.05
Precipitation range	0.81	<0.001	0.05	0.01
Precipitation seasonality	**0.95**	**<0.001**	0.07	0.01
**TOPOGRAPHIC VARIABLES**				
Minimum elevation	-0.01	0.44	**-0.96**	**<0.001**
Maximum elevation	**-0.90**	**<0.001**	-0.12	<0.001
Elevational standard deviation	-0.80	<0.001	0.48	<0.001
Slope range	0.76	<0.001	0.17	<0.001
Slope standard deviation	0.70	<0.001	0.15	<0.001
Aspect standard deviation	0.10	<0.001	0.14	<0.001
**VEGETATION STRUCTURE**				
Moist forests	**0.99**	**<0.001**	-0.06	0.005
Dry forests	-0.82	<0.001	-0.53	<0.001
Várzea	-0.43	<0.001	**0.90**	**<0.001**
Savannas and Shrublands	-0.002	0.93	-0.02	0.22
Mangroves	-0.07	0.002	-0.02	0.33
Montane forest	-0.10	<0.001	-0.08	0.002
